# SLO-Net: Enhancing Multiple Sclerosis Diagnosis Beyond Optical Coherence Tomography Using Infrared Reflectance Scanning Laser Ophthalmoscopy Images

**DOI:** 10.1167/tvst.13.7.13

**Published:** 2024-07-17

**Authors:** Roya Arian, Ali Aghababaei, Asieh Soltanipour, Zahra Khodabandeh, Sajed Rakhshani, Shwasa B. Iyer, Fereshteh Ashtari, Hossein Rabbani, Raheleh Kafieh

**Affiliations:** 1 Medical Image and Signal Processing Research Center, Isfahan University of Medical Sciences, Isfahan, Iran; 2 School of Medicine, Isfahan University of Medical Sciences, Isfahan, Iran; 3 Department of Engineering, Durham University, Durham, UK; 4 Isfahan Neurosciences Research Center, Isfahan University of Medical Sciences, Isfahan, Iran

**Keywords:** multiple sclerosis, optical coherence tomography, scanning laser ophthalmoscopy, artificial intelligence, deep learning

## Abstract

**Purpose:**

Several machine learning studies have used optical coherence tomography (OCT) for multiple sclerosis (MS) classification with promising outcomes. Infrared reflectance scanning laser ophthalmoscopy (IR-SLO) captures high-resolution fundus images, commonly combined with OCT for fixed B-scan positions. However, no machine learning research has utilized IR-SLO images for automated MS diagnosis.

**Methods:**

This study utilized a dataset comprised of IR-SLO images and OCT data from Isfahan, Iran, encompassing 32 MS and 70 healthy individuals. A number of convolutional neural networks (CNNs)—namely, VGG-16, VGG-19, ResNet-50, ResNet-101, and a custom architecture—were trained with both IR-SLO images and OCT thickness maps as two separate input datasets. The highest performing models for each modality were then integrated to create a bimodal model that receives the combination of OCT thickness maps and IR-SLO images. Subject-wise data splitting was employed to prevent data leakage among training, validation, and testing sets.

**Results:**

Overall, images of the 102 patients from the internal dataset were divided into test, validation, and training subsets. Subsequently, we employed a bootstrapping approach on the training data through iterative sampling with replacement. The performance of the proposed bimodal model was evaluated on the internal test dataset, demonstrating an accuracy of 92.40% ± 4.1% (95% confidence interval [CI], 83.61–98.08), sensitivity of 95.43% ± 5.75% (95% CI, 83.71–100.0), specificity of 92.82% ± 3.72% (95% CI, 81.15–96.77), area under the receiver operating characteristic (AUROC) curve of 96.99% ± 2.99% (95% CI, 86.11–99.78), and area under the precision–recall curve (AUPRC) of 97.27% ± 2.94% (95% CI, 86.83–99.83). Furthermore, to assess the model generalization ability, we examined its performance on an external test dataset following the same bootstrap methodology, achieving promising results, with accuracy of 85.43% ± 0.08% (95% CI, 71.43–100.0), sensitivity of 97.33% ± 0.06% (95% CI, 83.33–100.0), specificity of 84.6% ± 0.10% (95% CI, 71.43–100.0), AUROC curve of 99.67% ± 0.02% (95% CI, 95.63–100.0), and AUPRC of 99.65% ± 0.02% (95% CI, 94.90–100.0).

**Conclusions:**

Incorporating both modalities improves the performance of automated diagnosis of MS, showcasing the potential of utilizing IR-SLO as a complementary tool alongside OCT.

**Translational Relevance:**

Should the results of our proposed bimodal model be validated in future work with larger and more diverse datasets, diagnosis of MS based on both OCT and IR-SLO can be reliably integrated into routine clinical practice.

## Introduction

Multiple sclerosis (MS) is an autoimmune disease of the central nervous system characterized by chronic inflammation, demyelination, and axonal degeneration.^1^ Currently, a diagnosis of MS is based on clinical presentations, magnetic resonance imaging (MRI) findings, and the presence of oligoclonal bands in the cerebrospinal fluid (CSF).^2^ Notably, optical coherence tomography (OCT) studies have shown that the peripapillary retinal nerve fiber layer (RNFL) and both ganglionic cell and inner plexiform layers in the macular region (collectively abbreviated as mGCIPL) are thinner in MS patients compared with healthy controls (HCs)^3^; a retrograde neuroaxonal atrophy following acute inflammatory attacks could be a likely explanation.^4^ The thickness of these layers has been associated with patients’ visual problems, MS subtypes, physical and cognitive disability, and MRI findings.^5^ Therefore, OCT parameters are now regarded as useful biomarkers for the quantitation of neurodegeneration in MS, allowing for facilitated monitoring of disability progression and assessing the efficacy of neuroprotective therapies.^4^ Artificial intelligence (AI) has emerged as a promising aid for diagnosing MS,^6^ with impressive performance being shown in a recent meta-analysis.^7^ Data analyzed for the automated classification of MS primarily stem from MRI, serum, CSF, and OCT investigations^8^; specifically, the OCT parameters have involved the macular and/or peripapillary thickness of RNFL, GCIPL, inner nuclear layer (INL), and the whole retina, alone or in combination,^9^^–^^20^ leading to high levels of accuracy (ACC) reaching up to 100%.^14^

**Figure 1.**
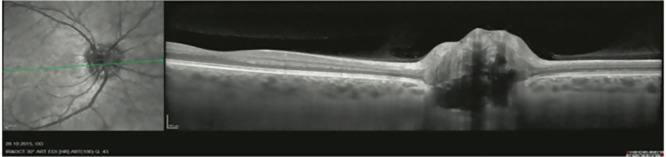
The acquisition window of the SPECTRALIS software containing both the IR-SLO image (*left*) and the OCT B-scan (*right*). Note that the *green line* superimposed on the IR-SLO image corresponds to the position of the B-scan. (Reprinted from Aumann et al.^23^ under the terms of the Creative Commons Attribution 4.0 International License.^24^)

Infrared scanning laser ophthalmoscopy (IR-SLO), also known as monochromatic fundus imaging, is another widely used retinal imaging technology that uses low-powered laser light to create two-dimensional images of the retina. IR-SLO is usually performed along with OCT B-scan acquisition; this approach allows for accurate alignment of the B-scans despite eye movements, which improves the signal-to-noise ratio and reduces measurement variability at follow-up examinations^21^ (Fig. 1). Compared to fundus camera images that share a similar appearance, IR-SLO images pose different contrast characteristics, meaning that some structures that are not obvious on fundus photographs may show up well using IR-SLO images; conversely, there could be imperceptible regions in IR-SLO images that fundus photography captures well. This is because each imaging modality uses varied wavelengths.^22^

Currently, machine learning–based diagnosis of MS relies solely on OCT thickness measurements.^7^ One underlying reason for this could be the fact that evidence on retinal pathology is primarily obtained from structural OCT investigations; however, a number of studies have shown that the retinal vascular system is also damaged in MS.^25^ According to a recent systematic review and meta-analysis of OCT angiography (OCTA) studies, including 1552 MS and 1107 HC eyes, the vessel density of the superior and deep capillary plexuses within different peripapillary and macular regions is significantly decreased in patients with MS compared with HC individuals.^25^ Of note, retinal imaging modalities, such as fundus camera photography and IR-SLO, that provide en face pictures of the retina typically lack structural and/or vascular pathological changes that can be readily identified by human physicians; therefore, it is of relevance to take advantage of machine learning models to investigate whether features capable of distinguishing between MS and HC states can be detected within these images.

In the current study, we aimed to develop deep learning (DL) models for classifying MS based on IR-SLO images using a dataset provided in a previous work.^26^ To our knowledge, this is a pioneering study incorporating IR-SLO into automated diagnosis of MS. Moreover, our work is among the few studies that have applied DL^14^^,^^20^^,^^27^ to retinal imaging data for detecting MS.

## Materials and Methods

### Dataset

#### Internal Dataset

In this study, we utilized 265 IR-SLO images from 32 patients with MS and 70 HC individuals, captured using a SPECTRALIS SD-OCT device (Heidelberg Engineering, Heidelberg, Germany). The dataset was obtained from a previous study by Ashtari et al.,^26^ performed between April 2017 and March 2019 at Kashani Comprehensive MS Center, Isfahan, Iran, a main referral center for MS in Isfahan.

All OCT B-scans were examined using Heidelberg Eye Explorer (HEYEX) version 5.1 (Heidelberg Engineering) and were controlled for a sufficient quality according to the OSCAR-IB criteria^28^ by an experienced technician. By applying the automatic real time mean algorithm, the scans were repeatedly captured in the same location nine times and then averaged, thereby reducing both the speckle noise and fluctuations in the background noise.^23^ The B-scans were segmented into nine boundaries using a graph-based method that relies on regional texture features^29^; all segmented scans were then checked by an expert and manually corrected whenever needed. Macular OCT volumes corresponding to a 6 × 6-mm area centered around the fovea, consisting of 45 horizontal B-scans, each composed of 512 A-scans, with an axial resolution (between 2 pixels in the A-scans) of 3.8 µm, were finally saved into a .vol file.

#### External Dataset

To investigate the generalization ability of our proposed model, an external dataset from an independent center (Johns Hopkins University, here referred to as the Johns Hopkins dataset) was also utilized. A detailed comparison of the demographic characteristics of the internal and external datasets is provided in Table 1. The Johns Hopkins dataset employed in this study consisted of IR-SLO images and OCT data from the right eyes of 32 individuals (14 HC and 18 MS). The dataset was collected using the SPECTRALIS SD-OCT device (Heidelberg Engineering), and all B-scans were segmented into nine boundaries using the internally developed software. Similar to the Isfahan dataset, macular OCT volumetric data covered an area of 6 × 6 mm around the fovea, consisting of 49 B-scans, each composed of 1024 A-scans, with an axial resolution of 3.9 µm. In order to match the contrast and brightness of the external dataset to our internal data, we increased the contrast by a factor of 1.25 and adjusted the brightness by 40 units for IR-SLO images. Additionally, for OCT thickness maps, we reduced the contrast by a factor of 0.70 and applied a brightness parameter of 40. The IR-SLO images and OCT thickness maps from both datasets were resized to 128 × 128 × 1 and 60 × 256 × 1 pixels, respectively. Also, images that belonged to the left eyes in the Isfahan dataset were mirrored to achieve uniform orientation across all images.

**Table 1.** Demographic Characteristics of the Participants in the Isfahan and Johns Hopkins Datasets

		Isfahan (*n* = 102)^a^	Johns Hopkins (*n* = 35)	*P* ^b^
Mean age ± SD	MS	34.13 ± 8.53	41.97 ± 8.77	0.002*
	HC	31.87 ± 7.66	35.77 ± 13.03	0.678
	All	32.58 ± 7.97	39.49 ± 10.94	0.001*
Gender (female/male)	MS	32/0	17/4	0.042*
	HC	53/15	12/2	0.771
	All	85/15	29/6	0.976

a For the Isfahan dataset, there were missing values for two individuals’ gender; therefore, descriptive analysis for gender was performed for 100 individuals.

b The statistical comparisons between the Isfahan and Johns Hopkins datasets were implemented using independent Students’ *t*-test or its non-parametric equivalent (i.e., Mann–Whitney *U* test), if the data did not follow a normal distribution (according to the Kolmogorov–Smirnov test) and χ^2^ tests.

* *P* < 0.05 was considered statistically significant.

#### Training and Validation Data and Test Splitting

Initially, 20% of the subjects from the Isfahan dataset were selected to form the test dataset, enabling us to evaluate the generalization capability of our models on unseen images. For the remainder of the Isfahan dataset, random training and validation data splitting was performed using *k*-fold cross-validation (CV), where *k* was set as 5. The *k*-fold CV is preferred for random splitting in terms of completeness and generalization. It ensures that the system has seen the complete dataset for training and guarantees that both training and test sets on every observation of the dataset are selected an equal number of times (*k* − 1 times and 1 time, respectively); whereas, in random split by resampling, at each iteration duplicate members of the test set can be selected twice or even more. Thus, *k*-fold CV is the preferred choice over random data splitting because it guarantees that the entire dataset is used for training the model, and each observation appears an equal number of times (i.e., *k* − 1 times in training and 1 time in testing phases). On the other hand, random split may lead to duplicate selections in the test set due to resampling at each iteration.

Stratified sampling was also used to ensure that each fold had the same proportion of samples with a certain label (i.e., MS or HC). Furthermore, to prevent data leakage between the training and validation datasets, a “subject-wise” approach was followed that involves putting all images belonging to each individual, regardless of its left-or-right orientation, in a single group. Therefore, the images of the same participant could not be used in both training and validation datasets concurrently, preventing an overestimation of the model performance.^30^

#### Data Augmentation

Data augmentation is a popular preprocessing technique in machine learning studies with limited training data in order to minimize the risk of overfitting. It works by adding minor modifications to the original input images to create new but similar examples, artificially increasing the size and diversity of training samples. In this study, several geometric and color space transformations were applied to the IR-SLO images and thickness maps; each set of these transformations is specifically discussed in the relevant sections.

### Classification

#### Convolutional Neural Networks

DL is a broad term applied to machine learning algorithms that are based on deep neural networks (i.e., neural networks typically with three or more hidden layers). DL has gained much attention during recent years as it has yielded remarkable results in various applications, such as natural language processing, speech recognition, and computer vision. An important contributor to the high performance of DL-based algorithms in computer vision is convolutional neural networks (CNNs). The idea of CNNs is very similar to the way animal visual cortex processes the visual signals; lower level neurons capture simple features such as edges and corners, but higher level cells detect more complex patterns, such as shape and texture. Generally, CNNs consist of three types of layers: convolutional, pooling, and fully connected (FC). Training of a CNN involves updating the weights of convolutional and FC layers through the process of backpropagation so that the difference between the actual and predicted class is minimized.^31^

In this study, we took advantage of transfer learning by using a number of state-of-the-art CNN architectures—VGG-16,^32^ VGG-19,^32^ ResNet-50,^33^ and ResNet-101^33^—which have yielded high levels of classification ACCs on large image benchmarks such as ImageNet.^34^ The idea behind transfer learning is that, instead of learning from scratch, the knowledge learned by such high-performing architectures can be transferred to a new dataset with a much lower size, thereby preventing overfitting. In this study, the above architectures were utilized as a fixed feature extractor, where the weights of the models were frozen; however, the FC part was replaced by a custom one applicable to our binary classification task (MS vs. HC).

Also, the fine-tuning approach was applied to the CNN model with the best performance; the topmost convolutional layers that capture features specific to the new domain became unfrozen so that the weights of these layers could be updated during the training process. However, the first convolutional layers were still kept frozen because they detect general features shared between natural images found in the ImageNet dataset and the IR-SLO images. Furthermore, a custom CNN model was developed to be fully trained on IR-SLO images. In order to search for the optimal CNN hyperparameters, including learning rate, batch size, dropout probability, number of hidden layers of the FC part, and number of neurons in each hidden layer, the Optuna hyperparameter optimization framework was utilized.^35^

### Evaluation of Classification Models

The metrics that were employed to evaluate the model performance consisted of ACC, sensitivity (SE), specificity (SP), precision (PR), and F1 score, with the mathematical formula for calculating them being represented as follows:

(1) $$\begin{array}{c} \mathrm{ACC}=\;\frac{TP+TN}{TP+TN+FP+FN} \end{array}$$

(2) $$\begin{array}{c} \mathrm{SE}=\;\frac{TP}{TP+FN} \end{array}$$

(3) $$\begin{array}{c} \mathrm{SP}=\;\frac{TN}{TN+FP} \end{array}$$

(4) $$\begin{array}{c} \mathrm{PR}\;=\;\frac{TP}{TP+FP} \end{array}$$

(5) $$\begin{array}{c} F1\;=\;\frac{2\;\times \;TP}{2\;\times \;TP+FP+FN} \end{array}$$

where *TP*, *FN*, *TN*, and *FP* are true positive, false negative, true negative, and false positive, respectively. Moreover, the receiver operating characteristic (ROC) curves and precision–recall curves were plotted; the ROC curve illustrates the relationship between the true-positive and false-positive rates, and the precision–recall curve showcases the tradeoff between the precision and recall across various thresholds. The areas under these curves, known as the area under the ROC (AUROC) curve and area under the precision–recall curve (AUPRC), were also calculated. Moreover, the gradient-based class activation map (Grad-CAM)^36^ was utilized to obtain saliency heatmaps for interpreting CNN predictions.

Notably, a standard probability threshold of 0.5 was chosen for the evaluation metrics consisting of ACC, SE, SP, PR, and F1 score. Although other methods, such as Youden's *J* index,^37^ were also viable options, we found that utilizing a probability threshold of 0.5 yields better outcomes, particularly when ACC is selected for comparing the performance of the models. Additionally, the selection of ACC as the base metric for comparing the performance of different models is due to its intuitive appeal to non-technical audiences, ease of interpretation, and lack of bias in datasets where class imbalance is absent, such as the one in this study. Although other metrics such as sensitivity would take precedence in designing research with specific purposes (e.g., screening to ensure individuals with MS are not missed and to minimize false negatives), this choice does not alter the analysis or methodological approach outlined in this manuscript and can be selected in different versions of this study.

All of the experiments in this study were implemented using Python programming language in the Keras platform backend in Python 3.7 software environment (code and models are available at https://github.com/royaarian101/SLO-MSNet).

## Results

Initially, we demonstrated the effectiveness of IR-SLO in the diagnosis of MS. However, considering that IR-SLO images are acquired using the same devices as OCT images, we proposed utilizing IR-SLO images as a supplementary tool to enhance the diagnostic potential of OCT data. To achieve this, OCT thickness maps of the entire retina were also incorporated as input data, and the performance of each modality was assessed in comparison to the combined approach.

The entire dataset was comprised of 132 IR-SLO and 124 OCT images from HC individuals and 133 IR-SLO and 60 OCT images from patients with MS. The variation in the number of OCT thickness maps and IR-SLO images is due to certain cases undergoing multiple imaging sessions due to data quality issues in each modality. Despite this, we included all high-quality images, even if one modality was excluded. For all three subsequent sections (two unimodal models with IR-SLO and OCT thickness maps and one bimodal with a combination of IR-SLO and OCT thickness maps), test data were chosen and set aside for final analysis, consisting of 27 IR-SLO and 29 OCT images from HC individuals and 24 IR-SLO and 14 OCT images from patients with MS. Subsequently, in each fold, after splitting the training and validation datasets, the IR-SLO training images underwent the augmentation process with a rotation range of ±5, width shift range of (–30, 30), height shift range of (–5, 5), zoom range of ±0.2, brightness range of (0.2, 1.5), and vertical flip. Similarly, augmentation of OCT thickness images of the training dataset, involved a rotation range of ±10, width shift range of (–30, 30), and zoom range of ±0.2.

Of note, the imbalance between the number of MS and HC OCT thickness maps was addressed during the training and evaluation phases. We oversampled the minority class by employing augmentation techniques, including rotation, zoom, and flipping. Also, robust performance metrics, such as F1 score and AUROC curve, were used for a more accurate assessment.

### Classification of MS Using IR-SLO Images

**Figure 2.**
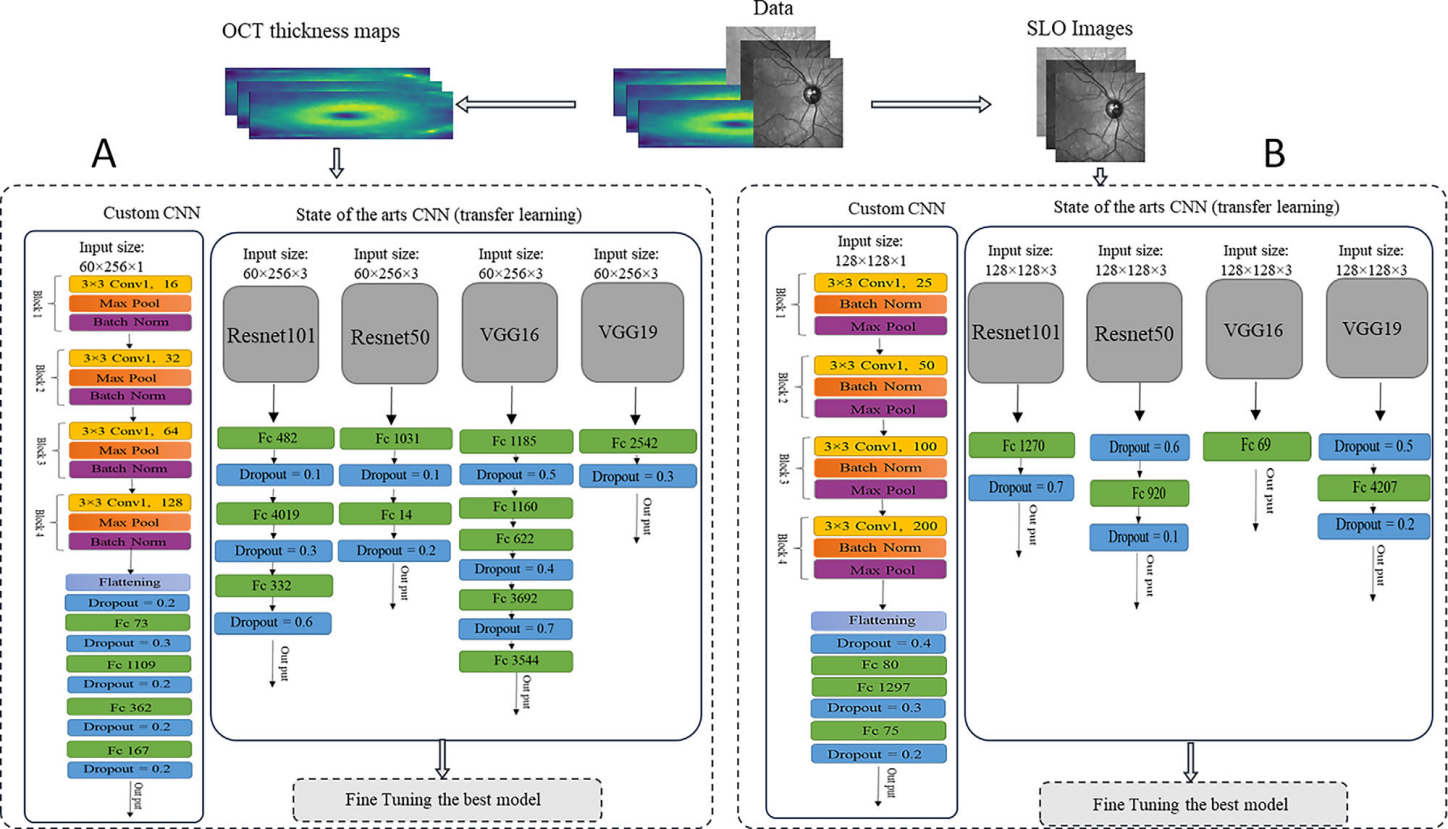
An overview of the models utilized for (**A**) IR-SLO images and (**B**) OCT thickness maps.

**Figure 3.**
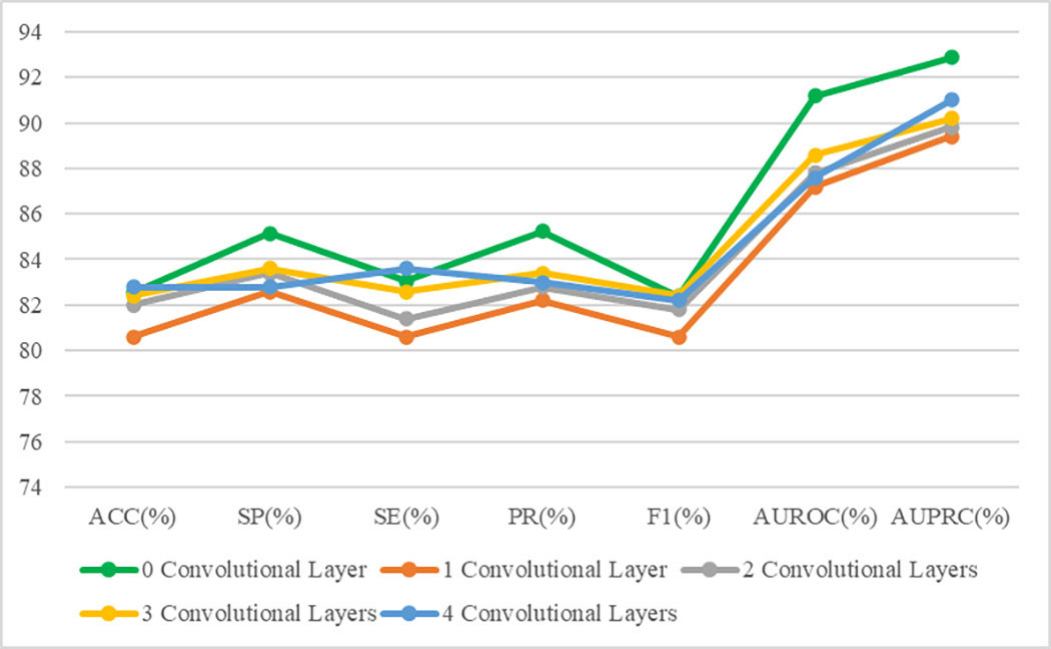
Performance metrics obtained after fine-tunning ResNet-101 so only SLO images were utilized for training.

After an initial random selection of 56 images (HC = 27, MS = 29) as the test dataset, the remaining images were finally utilized as training and validation datasets, using stratified *k*-fold CV. Figure 2A illustrates the architecture of the models used for IR-SLO images. As shown in Table 2, the CNN with a backbone of ResNet-101 yielded the most appealing results (ACC = 82.57% ± 0.42%, SE = 83.06% ± 1.62%, SP = 85.14% ± 3.82%, AUROC curve = 91.19% ± 0.57%, AUPRC = 92.87% ± 0.39%). To further improve the results, we also unfroze the first, second, third, and fourth topmost convolutional layers of ResNet-101, so additional numbers of parameters could be retrained with the IR-SLO image dataset. However, this fine-tuning approach did not lead to a better performance. For example, when the first topmost layer was unfrozen, the results obtained were as follows: ACC = 80.6%, SE = 80.6%, SP = 82.6%, AUROC curve = 87.2%, and AUPRC = 89.4% (Table 3, Fig. 3).

**Table 2.** Performance Metrics of Models Where Only SLO Images Were Utilized

	Mean ± SD							Optimal Hyper Parameters	
Model	ACC (%)	SP (%)	SE (%)	PR (%)	F1 Score (%)	AUROC Curve (%)	AUPRC (%)	Batch Size, *n*	Learning Rate
ResNet-50	75.00 ± 3.09	76.97 ± 7.83	77.82 ± 2.40	80.15 ± 3.23	73.12 ± 4.60	80.45 ± 0.83	87.28 ± 0.39	8	1.6e-4
ResNet-101	82.57 ± 0.42	85.14 ± 3.82	83.06 ± 1.62	85.25 ± 3.45	82.39 ± 0.42	91.19 ± 0.57	92.87 ± 0.39	16	1.58e-4
VGG-16	80.78 ± 0.35	86.68 ± 2.23	78.91 ± 0.99	85.79 ± 1.96	80.63 ± 0.37	91.26 ± 0.31	92.71 ± 0.15	8	1.7e-4
VGG-19	80.71 ± 0.45	86.90 ± 0.63	78.74 ± 0.50	85.98 ± 0.67	80.55 ± 0.48	92.84 ± 0.54	93.76 ± 0.44	8	1.2e-4
Custom CNN	80.71 ± 0.50	82.89 ± 1.36	80.18 ± 1.35	80.50 ± 0.98	80.63 ± 0.54	89.88 ± 0.61	91.44 ± 0.50	16	1.34e-4

**Table 3.** Performance Metrics Obtained After Fine-Tunning the ResNet-101–Based Model So Only SLO Images Were Utilized for Training

SLO Data	ACC (%)	SP (%)	SE (%)	PR (%)	F1 Score (%)	AUROC Curve (%)	AUPRC (%)
0 convolutional layer	82.57	85.14	83.06	85.25	82.39	91.19	92.87
1 convolutional layer	80.6	82.6	80.6	82.2	80.6	87.2	89.4
2 convolutional layers	82	83.4	81.4	82.8	81.8	87.8	89.8
3 convolutional layers	82.4	83.6	82.6	83.4	82.4	88.6	90.2
4 convolutional layers	82.8	82.8	83.6	83	82.2	87.6	91

### Classification of MS Using OCT Thickness Maps

**Figure 4.**
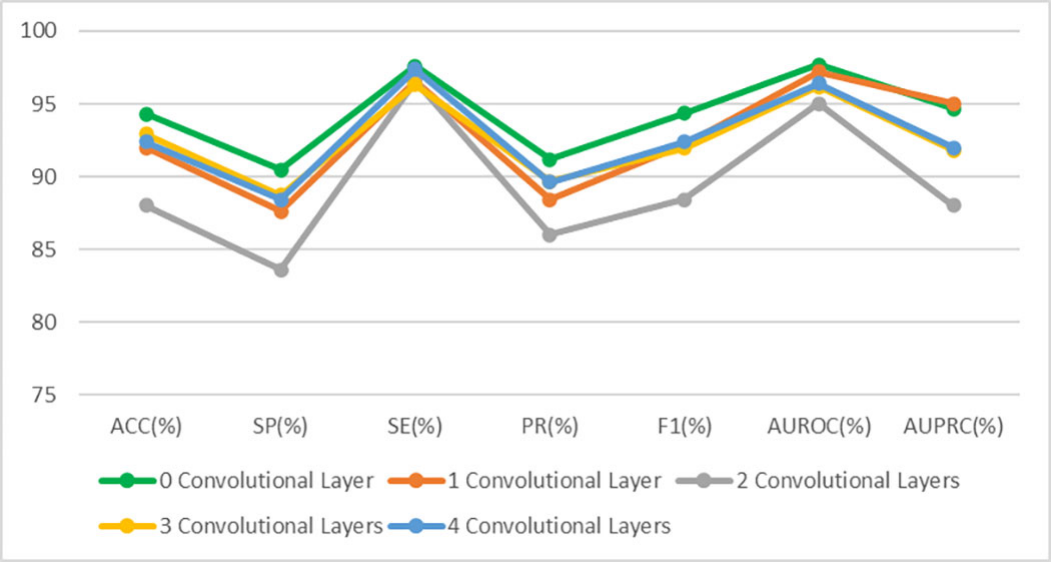
Performance metrics obtained after fine-tunning ResNet-101 so only OCT thickness maps were utilized for training.

In our next experiment, we aimed to investigate how model performance would change when OCT data were utilized instead of IR-SLO images. OCT thickness maps employed in this study were from the total retina and were calculated by subtracting the first and the last retinal boundaries. Figure 2B depicts the structures of the models developed for the training OCT thickness maps. Again, ResNet-101 emerged as the winning model (ACC = 94.32% ± 1.12%, SE = 97.59% ± 0.43%, SP = 90.48% ± 1.86%, AUROC curve = 97.70% ± 0.37%, AUPRC = 94.67% ± 1.03%) (Table 4). The fine-tunning experiments, performed in a similar manner as for the IR-SLO images, again did not lead to a better performance. For example, when the first topmost layer was unfrozen, the results obtained were as follows: ACC = 92%, SE = 96.6%, SP = 87.6%, AUROC curve = 97.2%, AUPRC = 95%) (Table 5, Fig. 4).

**Table 4.** Performance Metrics of Models Where Only OCT Thickness Maps Were Utilized

	Mean ± SD							Optimal Hyper Parameters	
Model	ACC (%)	SP (%)	SE (%)	PR (%)	F1 Score (%)	AUROC Curve (%)	AUPRC (%)	Batch Size, *n*	Learning Rate
ResNet-50	91.16 ± 1.38	85.42 ± 1.87	98.63 ± 0.52	87.36 ± 1.38	91.28 ± 1.36	95.05 ± 0.76	86.22 ± 1.82	32	4.34e-5
ResNet-101	94.32 ± 1.12	90.48 ± 1.86	97.59 ± 0.43	91.17 ± 1.57	94.34 ± 1.1	97.70 ± 0.37	94.67 ± 1.03	64	3.72e-4
VGG-16	92.32 ± 1.39	89.44 ± 0.61	95.68 ± 1.83	90.30 ± 0.65	92.32 ± 1.42	98.70 ± 0.19	97.86 ± 0.23	8	4e-4
VGG-19	90.53 ± 0.75	85.72 ± 0.92	95.79 ± 1.12	87.24 ± 0.73	90.63 ± 0.73	96.93 ± 0.53	95.16 ± 0.64	32	11.95e-5
Custom CNN	86.95 ± 1.47	83.92 ± 1.65	89.38 ± 1.41	85.51 ± 1.10	86.94 ± 1.50	92.10 ± 0.97	84.63 ± 2.22	16	13.36e-5

**Table 5.** Performance Metrics Obtained After Fine-Tunning the ResNet-101–Based Model So Only OCT Thickness Maps Were Utilized for Training

OCT Data	ACC (%)	SP (%)	SE (%)	PR (%)	F1 Score (%)	AUROC Curve (%)	AUPRC (%)
0 convolutional layer	94.32	90.48	97.59	91.17	94.34	97.7	94.67
1 convolutional layer	92	87.6	96.6	88.4	92.2	97.2	95
2 convolutional layers	88	83.6	96.6	86	88.4	95	88
3 convolutional layers	92.94	88.75	96.32	89.67	91.96	96.17	91.8
4 convolutional layers	92.4	88.4	97.4	89.6	92.4	96.4	92

### Classification of MS Using Both IR-SLO Images and OCT Thickness Maps

**Figure 5.**
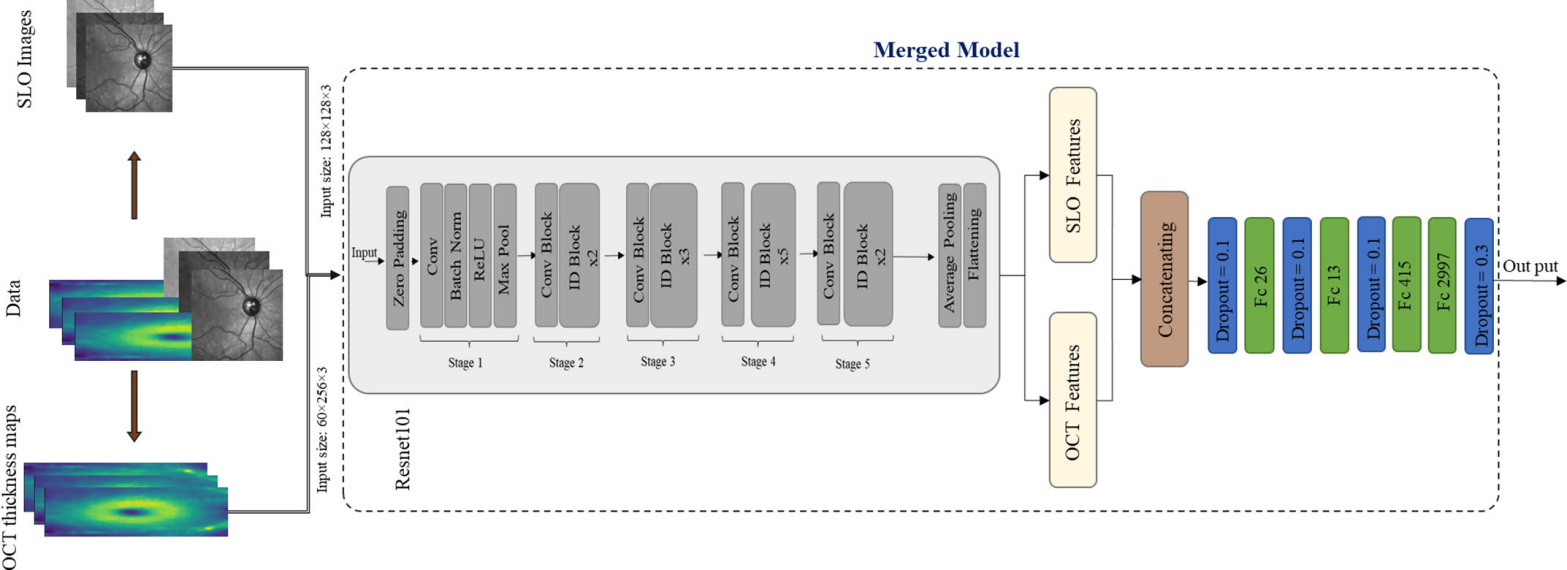
An overview of the bimodal model proposed in this study, where both SLO images and OCT thickness maps of the total retina were utilized to train a ResNet-101–based CNN.

In this step, we aimed to investigate whether adding IR-SLO images to OCT thickness maps leads to even superior classification performance. IR-SLO images were exclusively matched with OCT thickness maps of the corresponding eye. An overview of this merged model is illustrated in Figure 5. As ResNet-101 appeared to be the best-performing model for classifying MS based on both IR-SLO images and OCT thickness maps, it was selected as the convolutional part of the merged model with its weights being frozen. Freezing layers is justified as the test cases are precisely excluded in both methods using individual modalities (as described earlier), and the same test cases (number of cases in the test dataset was HC = 14 and MS= 7) are utilized in the combined model. The modality-specific features extracted by the convolutional part were then concatenated and given to a novel FC part. According to Table 6, the merged model was able to achieve superior performance compared to each of the models trained with either IR-SLO images or OCT thickness maps (ACC = 96.85% ± 0.45%, SE = 100% ± 0.0%, SP = 94.96% ± 0.66%, AUROC curve = 99.69% ± 0.12%, AUPRC = 99.75% ± 0.1%).

**Table 6.** Performance Metrics Obtained From Applying Subject-Wise, Eye-Wise, and Record-Wise Data-Splitting Approaches to the Best-Performing Model Trained With Both SLO Images and OCT Thickness Maps

	Mean ± SD							Optimal Hyper Parameters	
Data-Splitting Approach	ACC (%)	SP (%)	SE (%)	PR (%)	F1 Score (%)	AUROC Curve (%)	AUPRC (%)	Batch Size, *n*	Learning Rate
Subject-wise	96.85 ± 0.45	94.96 ± 0.66	100 ± 0.0	95.22 ± 0.60	96.83 ± 0.45	99.69 ± 0.12	99.75 ± 0.1	16	2.75e-4
Eye-wise	99.19 ± 0.45	99.85 ± 0.30	98.57 ± 0.11	99.86 ± 0.28	99.18 ± 0.44	99.99 ± 0.02	99.99 ± 0.01		
Record-wise	100 ± 0.0	100 ± 0.0	100 ± 0.0	100 ± 0.0	100 ± 0.0	100 ± 0.0	100 ± 0.0		

### Record-Wise, Eye-Wise, and Subject-Wise Data-Splitting Approaches

**Figure 6.**
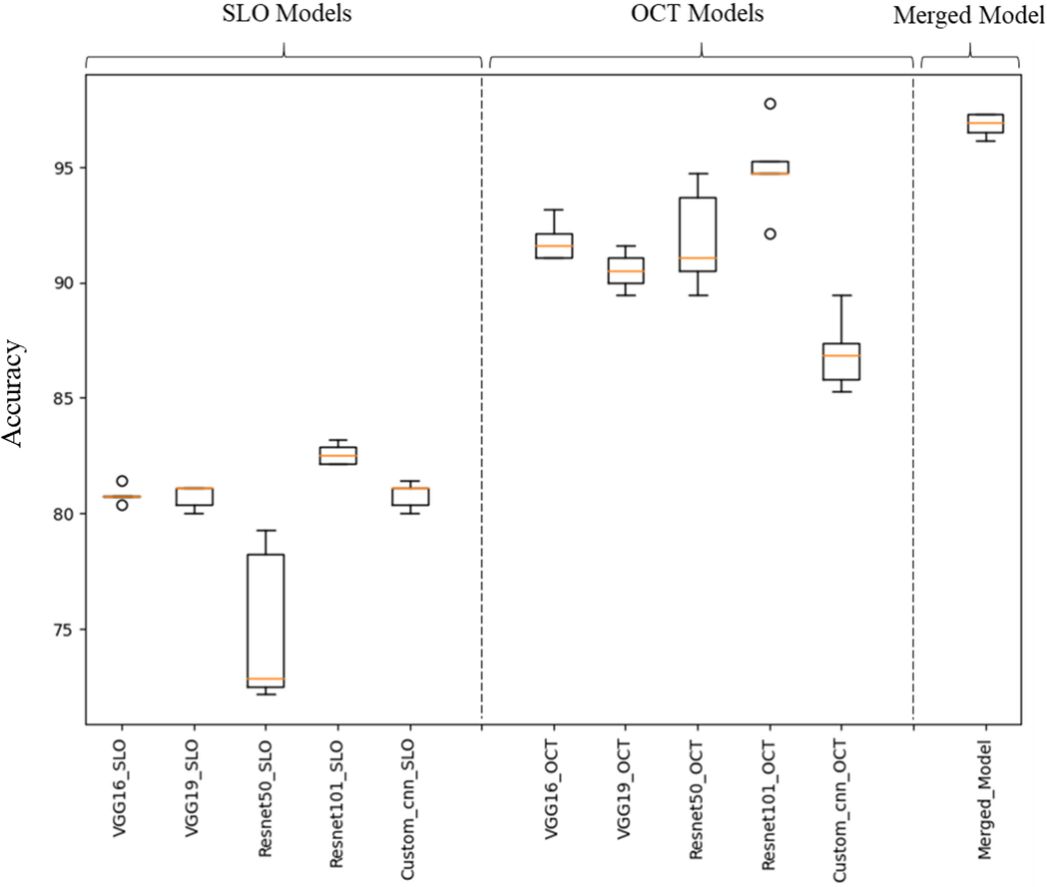
Box plots illustrating the variance in classification accuracy of different methods across five runs.

In addition to the subject-wise approach mentioned earlier, two other data-splitting methods were also applied: record-wise and eye-wise. The record-wise approach is a conventional data-splitting method in which the entire image dataset (MS class, 133 IR-SLO images and 60 OCT thickness maps; HC class, 132 IR-SLO images and 124 OCT thickness maps), after reserving 20% of the samples for the test dataset (acquired from seven MS cases and 14 HC cases), was given to the fivefold CV algorithm to create training and validation images in each fold. In the eye-wise approach, all images belonging to either the right or left eye of each individual were put in separate groups. This resulted in 118 (19 test and 99 training/validation datasets) and 59 (12 test and 47 training/validation datasets) “eye” groups for HC individuals and patients with MS, respectively. Results of these approaches are summarized in Table 6. Figure 6 presents a box plot showcasing the accuracy variations among the different methods across five runs.

### Bootstrapping

**Figure 7.**
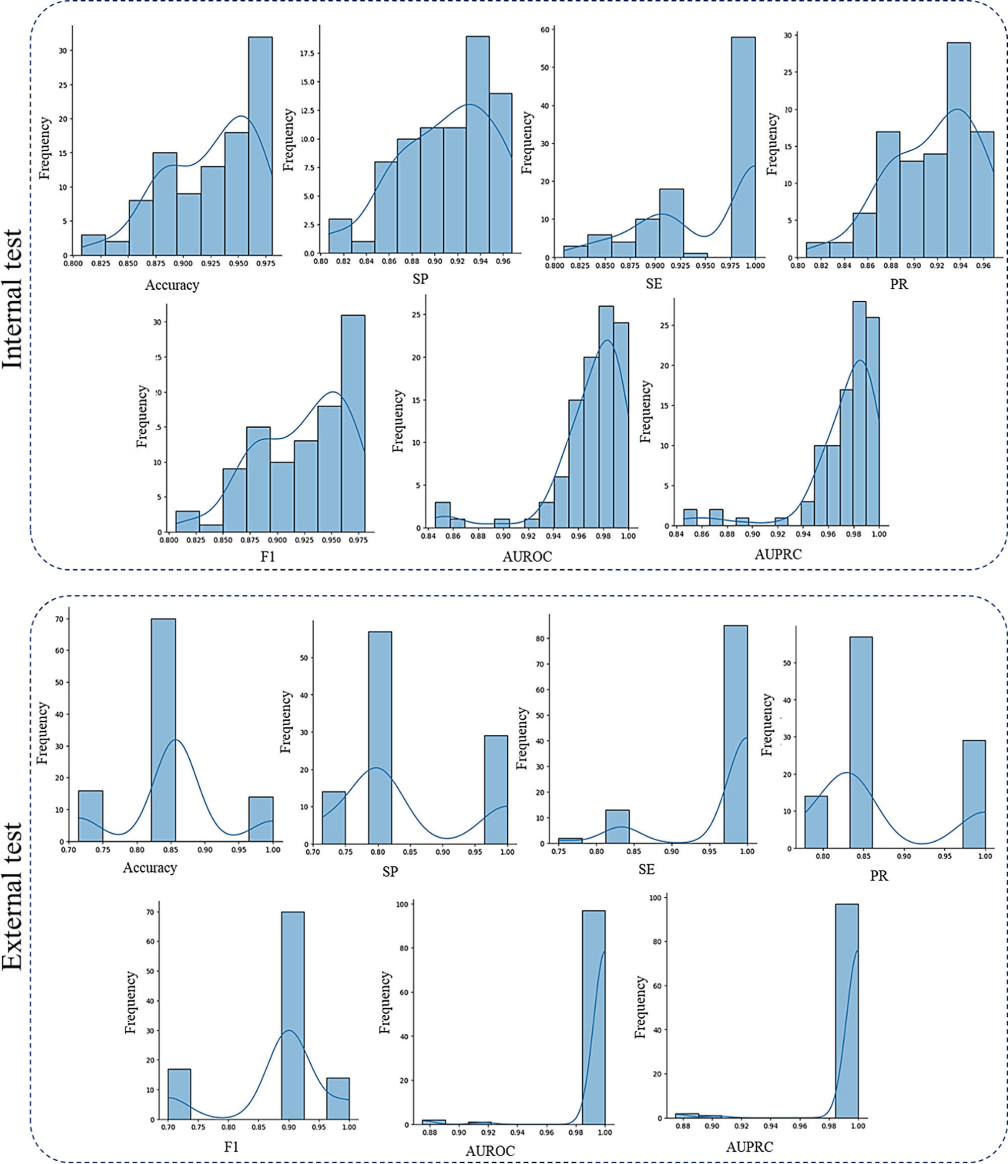
Histograms representing the results of both internal and external test datasets achieved by bootstrap aggregating of the best-performing CNN trained with both IR-SLO images and OCT thickness maps.

Bootstrap aggregating, commonly known as bagging, is a resampling technique fundamental to classification models, first introduced by Breiman in 1996.^38^ Through iterative sampling with replacement from the training dataset, it generates multiple subsets for training. This ensemble approach enhances model stability and generalization, mitigating issues associated with overfitting. To estimate confidence intervals for performance metrics such as ACC, a bootstrap procedure involves creating multiple subsets, training the model on each, and deriving a distribution of results. The confidence interval is then computed from this distribution, offering a robust measure of the model performance variability.^39^ Given the relatively small sample size of the dataset, one possible solution is to utilize bootstrapping with replacement and reporting the confidence interval. Table 7 and Figure 7 represent the results of bootstrapping for the proposed bimodal model.

**Table 7.** Performance Metrics Obtained by Bootstrap Aggregation of the Best Performing CNNs Trained With Both IR-SLO Images and OCT Thickness Maps

Test Data	ACC (%)	SP (%)	SE (%)	PR (%)	F1 Score (%)	AUROC Curve (%)	AUPRC (%)
Internal							
Mean ± SD	92.40 ± 4.1	92.82 ± 3.72	95.43 ± 5.75	91.62 ± 3.75	92.12 ± 4.35	96.99 ± 2.99	97.27 ± 2.94
95% CI	83.61–98.08	81.15–96.77	83.71–100.0	83.42–96.88	81.45–98.07	86.11–99.78	86.83–99.83
External							
Mean ± SD	85.43 ± 0.08	84.6 ± 0.10	97.33 ± 0.06	87.39 ± 0.08	84.95 ± 0.08	99.67 ± 0.02	99.65 ± 0.02
95% CI	71.43–100.0	71.43–100.0	83.33–100.0	77.78–100.0	70.23–100.0	95.63–100.0	94.90–100.0

### Generalization Ability of Our Models on an External Dataset

To check for the generalization ability of our optimal model (i.e., Res-101–based CNN trained with both IR-SLO images and OCT thickness maps of the Isfahan dataset), we used IR-SLO images and OCT thickness maps from the Johns Hopkins dataset (all of which belong to the right eye) as an extra source for the test phase. To achieve this goal, we divided the external data into the training, validation, and test sets with a ratio of 60:20:20, respectively. Subsequently, we fine-tuned the optimal model on the external training set through the bootstrapping approach. During this process, only the last convolutional layer was made trainable, and the others were kept frozen. Each iteration of the bootstrapping involved evaluating the newly trained model on the external test set. The outcomes are depicted in Figure 7 and Table 7.

### Model Interpretability

**Figure 8.**
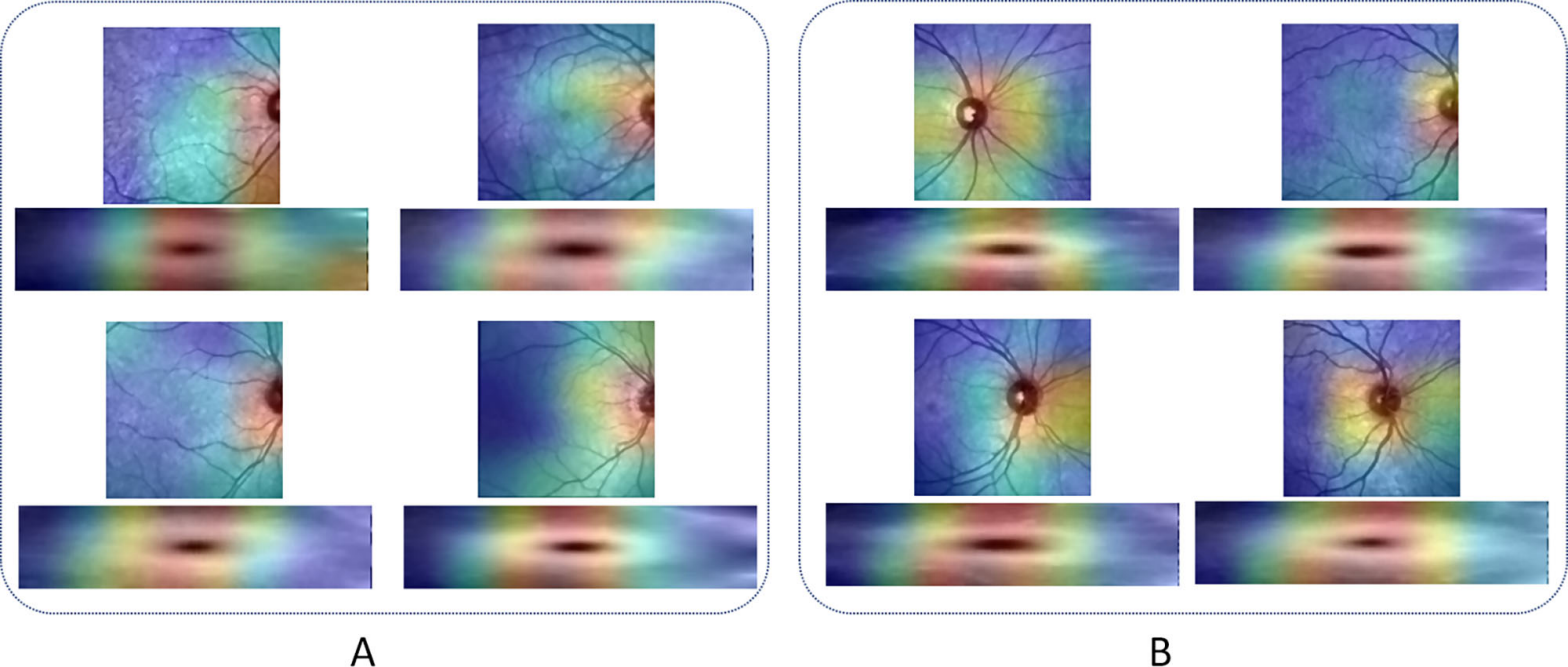
Saliency maps generated for eight SLO images and OCT thickness maps of the total retina of (**A**) HC and (**B**) MS subjects from the test dataset when the best-performing model trained with SLO images were utilized.

By applying the Grad-CAM algorithm to the best-performing model trained with IR-SLO images, the saliency maps visualized in Figure 8 were generated.

## Discussion

The incorporation of OCT technology with IR-SLO in SPECTRALIS SD-OCT devices allows for locking the B-scans at a desired position using a real-time eye tracking system (TruTrack), which removes the effects of eye motion during image acquisition, creating high-quality OCT images. Furthermore, this is necessary for an accurate evaluation of the disease progression, as a B-scan on the same position should be studied during follow-up examinations.^23^ Given that IR-SLO images are also available when OCT scans are being captured, we examined whether any additional features can be provided from the IR-SLO images, which would be useful for discriminating between MS and HC individuals.

Notably, we showed that the model trained solely with OCT thickness maps outperformed the model that relied only on IR-SLO images, suggesting that IR-SLO images may lack sufficient features for distinguishing between MS and HC subjects. However, integration of IR-SLO images and OCT thickness maps led to superior model performance (approximately 3% higher than when only OCT thickness maps were utilized). This is indeed in line with our expectations, as the merged model leverages a broader range of input images from two distinct modalities, thus incorporating more useful information for accurate detection of MS. In addition, among CNNs that were trained with either IR-SLO images or OCT thickness maps, those with a backbone of ReNet-101 led to the most promising results, revealing that complex models with much greater number of parameters are necessary to extract features from IR-SLO and OCT thickness maps that are appropriate for distinguishing between MS and HC.

According to Table 6, as expected, the best results were achieved using the record-wise approach, followed by eye-wise and subject-wise data-splitting methods. In both record-wise and eye-wise approaches, it is possible to simultaneously utilize images of a participant as the training data and the other images belonging to the same person as the validation data; therefore, the subject-wise method seems to be a more reliable data-splitting approach. This is because this strategy is potentially protected against an overestimation of the model performance due to the absence of data leakage between the training and validation datasets.^30^ As shown in Table 6, should a conventional record-wise approach be employed, a best ACC of 100% would be achieved, which is higher than that of most of the OCT studies, the majority of which have used the same technique for training and validation data splitting. The only exception that employed a subject-wise approach is the study by Khodabandeh et al.,^18^ who used the Isfahan dataset, similar to the current study. This allowed us to directly compare the results of these two studies; however, Khodabandeh et al.^18^ relied solely on OCT scans. They cropped squares 20 × 20, 30 × 30, and 40 × 40 pixels around the macula from the OCT thickness maps of different retinal layers and applied principal component analysis (PCA) and recursive feature elimination for dimensionality reduction; finally, three conventional machine learning classifiers—support vector machine (SVM), random forest, and neural networks—were trained with the obtained features. The authors were able to reach an ACC of 88% using an SVM with a linear kernel that was applied to the PCA-extracted features from the GCIPL/INL thickness maps. In the current study, we showed that ACC = 96.85% ± 0.45%, SE = 100% ± 0.0%, SP = 94.96% ± 0.66%, AUROC curve = 99.69% ± 0.12%, and AUPRC = 99.75% ± 0.1% can be achieved when CNN-based models are trained with OCT and IR-SLO data.

As illustrated in Figure 8, the optic nerve head (ONH) and the area around are where our IR-SLO–based models focused to distinguish between MS and HC state. Optic nerve neuropathy, with or without clinical symptoms, is observed in nearly all patients with MS, as confirmed by postmortem pathological investigations.^4^ During an ophthalmologic examination, a clinician is able to diagnose RNFL damage in optic neuritis but only when more than 50% of this layer has been destroyed.^40^ This highlights the important role that an AI-based system can play, as demonstrated in the current study, where the model identified minor optic nerve pathologies that may not be readily recognizable by humans within an en face image such as IR-SLO. Furthermore, the proposed model has also focused on the vessels around the ONH, which is in line with previous OCTA studies where the ONH blood flow index, defined as the average flow signal within the en face OCTA image, has shown to be reduced in MS patients, both with and without optic neuritis, compared to HC individuals.^25^^,^^41^^,^^42^ OCTA studies have also shown a decreased level of radial peripapillary capillary vessel density within superior, nasal, and temporal sectors in patients positive for optic neuritis compared to patients who are not.^25^ Alterations of retinal blood flow in MS have been linked to a decrease in metabolic demand due to ganglionic cell degeneration as a result of the neuroinflammatory mechanisms.^25^ Conversely, some studies have hypothesized that retinal vascular pathology serves as a primary trigger, causing hypoxia, which in turn gives rise to inflammation and neurodegeneration.^43^^,^^44^ In addition, as is shown within heatmaps of OCT thickness maps, the area around the fovea has been identified as an important region for discriminating between MS and HC individuals, in line with previous studies showing reductions in thickness of the total macula, macular RNFL, or GCIPL in MS patients.^4^^,^^45^

During recent years, automated diagnosis of MS has been made possible using machine learning algorithms, with a remarkable overall ACC of 94%.^7^ Various input data have been utilized thus far, with the most desirable results achieved using the parameters obtained from MRI (pooled ACC = 96%), OCT (pooled ACC = 93%), CSF/serum (pooled ACC = 93%), and even gait and breathing pattern (pooled ACC = 88%) investigations. The OCT-based studies have mainly applied conventional machine learning classifiers on the thickness values^9^^,^^10^^,^^12^^,^^13^^,^^15^^–^^17^^,^^19^ or the extracted features from them,^11^^,^^14^^,^^18^ with only two studies utilizing DL.^14^ López-Dorado et al.^14^ employed Cohen's *d* coefficient technique on the thickness maps of RNFL, ganglion cell layer (GCL^+^; equivalent to GCIPL), GCL^++^ (equivalent to GCIPL plus RNFL), the total retina, and the choroid from 48 MS patients and 48 HC individuals. The resulting thickness map images were then given to a custom CNN model made up of two successive blocks, each containing one convolutional and one pooling layer, ultimately achieving very encouraging results (SE = 100%, SP = 100%). This finding is akin to our results when a similar training and test data splitting approach (record-wise) was employed. Similar to the study by López-Dorado et al.,^14^ three other studies captured OCT data using a swept-source device (SS-OCT),^9^^,^^10^^,^^12^ all leading to ACC levels of more than 90%. Such promising results could partly be attributed to the high-resolution scans generated by the SS-OCT technology. It should be noted that these studies had a limited sample size with insufficient diversity; indeed, three studies used the same dataset.^9^^,^^12^^,^^14^ In the second study that utilized DL for distinguishing between MS and HC, Ortiz et al.^27^ first analyzed the AUROC curve of the average thickness of both eyes and the inter-eye thickness differences for each of the nine segmented retinal layers (RNFL, GCL, IPL, INL, outer plexiform layer [OPL], outer nuclear layer [ONL], retinal pigment epithelium [RPE], inner retinal layers [RNFL, GCL, IPL, INL, OPL, ONL], and outer retinal layers [RPE and photoreceptors layer]), and identified the most important features accordingly. The GCL average thickness and IPL inter-eye thickness differences were finally selected to be used for training a CNN from scratch. The input size was 8 × 8 × 2, and the model architecture consisted of two consecutive convolutional layers with 16 and 32 kernels (with a size of 3 × 3), generating a 4 × 4 × 32 feature map, which was given to a FC network at the end; an ACC of 87%, a SE of 82%, and a SP of 92% were finally achieved.^27^ The largest study that aimed to classify MS based on OCT data was undertaken by Kenney et al.,^19^ who evaluated 1568 MS patients and 552 HC subjects from the United States, Europe, and the Middle East. The dataset included various demographic, visual acuity, and SD-OCT parameters; using classification and regression tree models, the authors identified GCIPL thickness of both eyes on average, inter-eye GCIPL thickness difference, and binocular 2.5% low-contrast letter acuity as the features with the highest discriminant capacity. Kenney et al.^19^ applied both logistic regression and SVM algorithms that ultimately were shown to have a similar performance. The use of SVM with a linear kernel achieved an ACC of 88%, a SE of 83%, and a SP of 90%. Overall, although the majority of machine learning research on MS classification has taken advantage of MRI,^8^ OCT measurements have also been shown to be invaluable input data. Notably, the diagnostic performance of the models trained with MRI and OCT parameters are not far different, but the OCT technology is much less invasive and costly. In the current study, we utilized IR-SLO images in addition to OCT data, resulting in best ACCs of 100% ± 0.0%, 99.19% ± 0.45%, and 96.85% ± 0.45%, respectively, for record-wise, eye-wise, and subject-wise data-splitting approaches. As mentioned above, the proposed bimodal model is indeed a ResNet-101–based CNN with novel FC architecture fitted to our dataset.

This study is the first work in which machine learning models were trained with two different imaging modalities for the diagnosis of MS. Unlike MS, in previous studies on neurodegenerative diseases such as Alzheimer's disease and Parkinson's disease, fundus camera photographs, OCT angiography, and ultra-widefield color and fundus autofluorescence SLO images have been utilized as input data to classification models. For example, Wisely et al.^46^^,^^47^ trained a RenNet50-based CNN with data obtained from OCT, OCTA, and ultra-widefield color and fundus autofluorescence SLO to distinguish patients with mild cognitive impairment^46^ or Alzheimer's disease^47^ from HC individuals, with AUROC curves of 0.81 and 0.84, respectively, when applied to the test set. Two studies on patients with Parkinson's disease have also been able to detect Parkinson's disease^48^ or differentiate it from movement disorders atypical for Parkinson's disease^49^ based on fundus camera photographs, yielding encouraging performance of 71% and 70% ACCs, respectively.

This study had several limitations. Initially, the internal dataset originated from a single center and was comprised of a limited number of samples, thus limiting the generalization ability of our models to real-world scenarios. To address this limitation, we employed various data augmentation techniques to generate similar input data with slight variations, thereby increasing the number of images used for training. Additionally, we utilized the bootstrapping method, demonstrating that our top-performing model maintained strong performance across 100 runs involving different combinations of training samples. Furthermore, we validated the classification performance of our proposed model on an external independent dataset and achieved acceptable results. Second, we were not able to separate the eyes with a prior history of optic neuritis (ON), which are shown to have a thinner RNFL and GCIPL compared to ON-negative eyes. Hypothetically, IR-SLO images of the ON-positive eyes might also become more affected and have to be separated similar to OCT maps to have more robust results. Third, a reliable statistical comparison of the performance of the different models was not feasible. Indeed, the independence assumption fundamental to statistical hypothesis tests was not at all met, as all of the models were repeatedly applied to the same test dataset across different folds and iterations; the assumption was even further violated because the images within each of the training, validation, and test datasets lacked complete independence, given that both images of an individual's eyes were utilized through our subject-wise data-splitting approach. Finally, the cross-sectional nature of this study precludes any conclusion regarding the disease progression.

## Conclusions

To conclude, we have taken a significant step toward automated and precise detection of MS using a non-invasive, low-cost, and easily accessible technology. This is of great importance, as in current clinical practice diagnosing MS is a challenging and time-consuming task that relies heavily on the findings from MRI and CSF investigations. We showed that a hybrid CNN receiving input data from both modalities can detect MS with astonishing ACCs near 100%. In order to enhance the reliability and real-world applicability of our findings, we employed a subject-wise training and validation data-splitting strategy during *k*-fold cross-validation. Future studies can incorporate IR-SLO images with fundus camera photographs or other types of OCT data such as thickness maps of RNFL and GCIPL or projection images, as well. Projection images are typically generated by taking the average of OCT A-scan intensities between the retinal layer boundaries.^50^ With this increased variety of information given to the model, more favorable outcomes could potentially be attained. Indeed, large-scale multicenter studies are encouraged to further evaluate the diagnostic ACC of machine learning algorithms trained with OCT thickness maps and IR-SLO images, paving the way for their integration into routine clinical practice.
